# Factors contributing to flares of ulcerative colitis in North India- a case-control study

**DOI:** 10.1186/s12876-023-02978-y

**Published:** 2023-09-28

**Authors:** Vishavdeep Singh Rana, Gaurav Mahajan, Amol N. Patil, Anupam K. Singh, Vaneet Jearth, Aravind Sekar, Harjeet Singh, Atul Saroch, Usha Dutta, Vishal Sharma

**Affiliations:** 1 Department of Internal Medicine, Postgraduate Institute of Medical Education and Research, Chandigarh, India; 2 Department of Gastroenterology, Postgraduate Institute of Medical Education and Research, Chandigarh, India; 3 Department of Pharmacology, Postgraduate Institute of Medical Education and Research, Chandigarh, India; 4 Department of Histopathology, Postgraduate Institute of Medical Education and Research, Chandigarh, India; 5 Department of Surgical Gastroenterology, Postgraduate Institute of Medical Education and Research, Chandigarh, India

**Keywords:** Inflammatory bowel disease, Crohn’s disease, Ulcerative colitis, Stress, Antibiotics, NSAIDs, Adherence, Cost

## Abstract

**Background:**

Ulcerative colitis is a relapsing and remitting disease that may be associated with flares. The causes of flares in the Indian setting are not well recognized.

**Methods:**

The present prospective case-control study was conducted at a single center in North India. Cases were defined as patients admitted for flare of ulcerative colitis, while controls were patients in remission enrolled from the outpatient department. The basis of the diagnosis of flare was a simple clinical colitis activity index (SCCAI) of ≥ 5 and endoscopic activity, while remission was based on SCCAI < 4 and a normal fecal calprotectin. A questionnaire evaluating recent infections, stress, drug intake (antibiotics, pain medication), adherence to therapy, and use of complementary and alternative therapy (CAM) was administered.

**Results:**

We included 84 patients (51 with flare and 33 in remission) with a median age of 38 years, of whom 47 (55.9%) were males. The two groups were similar for baseline parameters, including age (38, 23–50 and 38, 25.5–48.5 years), male gender (52.9% and 60.6%), extent of disease, extraintestinal manifestations (21.6% and 12.1%), use of 5-aminosalicylates (76.5% and 90.9%). The thiopurine use was lower in those having a flare (15.7% and 36.4%). Amongst the predictors of flare, the recent infections (39.2% and 30.3%), recent travel (31.4 and 27.3%), eating outside food (47.1% and 39.4%), consumption of milk products (88.2% and 75.8%), use of pain medication (43.1% and 33.3%) and recent stress (62.7% and 60.6%) were similar between cases and controls. The rates of antibiotic use (29.4% and 6.1%), lack of adherence (50.9% and 15.2%), and intake of CAM (70.6% and 33.3%) were higher in those with flare. Patients attributed a lack of adherence to the cost of therapy, presumed cure (due to lack of symptoms), and fear of adverse effects.

**Conclusion:**

Lack of adherence to inflammatory bowel disease therapies and recent CAM and antibiotic intake was higher in patients with flares of UC. The study makes ground for educational intervention(s) promoting knowledge and adherence to IBD therapies.

**Supplementary Information:**

The online version contains supplementary material available at 10.1186/s12876-023-02978-y.

## Introduction

Inflammatory bowel disease is increasing in South Asia and brings forth challenges in management that are unique to the region [1, 2]. Some of these challenges include difficulties in diagnosis (and differentiating from mimics), reliance on complementary and alternative medication (CAM) often promoted as ‘natural and safe’, and lack of penetrance of health care insurance which means that costs of the therapy for chronic diseases are often borne out of the pocket by patients [1]. Flares of IBD may necessitate additional investigations and intensification of therapy, which could increase the costs of care for patients in the region [3]. IBD flares could result from several factors, and certain additional modifiable factors are also recognized to play a role [4–12]. Previous reports have implicated several factors that could potentially result in IBD flares. These include lack of adherence to medication, infections (enteric infections, upper respiratory tract infections) in the preceding month, use of nonsteroidal anti-inflammatory agents (NSAIDs), life events, and stress in the preceding three months [4–12]. However, the factors significantly implicated in the flares vary between various studies, which suggests that the underlying factors for IBD flares may depend on the underlying disease (ulcerative colitis or Crohn’s disease) and the underlying population (geographic location, ethnicity) [6, 11–12].

This points to the need for regional information relevant to particular geographic regions to evaluate possible reasons for disease flares. Since the possible reasons for flare have not been systematically evaluated in India, we conducted a case-control study in North India to evaluate the reasons for disease flare in ulcerative colitis by comparing patients with flares to those in remission.

## Methods

### Setting

The study was conducted at a tertiary center in North India from 1st January 2022 to 30th November 2022. The study was conducted after obtaining ethical clearance from the Institutional Ethics Committee. A written informed consent before inclusion was obtained from the patients. We followed the ethical norms for human research as outlined in the Helsinki Declaration and the ICMR 2007 [13, 14].

### Patients

The cases were the patients with a flare of ulcerative colitis. We screened all patients with inflammatory bowel disease admitted during the study period for possible inclusion. The diagnosis of ulcerative colitis was established based on clinical, endoscopic, and histologic features as suggested in the European Crohn’s and Colitis Organization guidelines [15]. Those with underlying ulcerative colitis and admitted for a flare of ulcerative colitis were considered for inclusion. The flare of ulcerative colitis was defined based on two criteria- Simple Clinical Colitis Activity Index (SCCAI) score of 5 or more, which takes into account bowel frequency, nocturnal symptoms, urgency, blood in stools, general well-being, and presence of extraintestinal manifestations, and in addition, the presence of endoscopic activity (Mayo endoscopic subscore of > 1) [16, 17]. We excluded patients who were admitted for reasons other than bowel disease flares (e.g. those with infections including gastrointestinal infections, extraintestinal complications, etc.), those with age 12 years or below, those with Crohn’s disease or IBD-unclassified, and those refusing consent for participation.

Controls were recruited from the outpatient department of the hospital. These were patients with ulcerative colitis diagnosed on the basis of standard criteria and deemed to be in disease remission with both (1) SCCAI of < 3 with stable bowel frequency over the past six months and (2) a normal fecal calprotectin (< 100 mcg/gram). In our IBD clinic, fecal calprotectin is tested in all patients every six months and more frequently amongst those who have active disease.

### Procedures

All patients with UC flares received standard evaluation (clinical history for the duration of symptoms, the type of symptoms, physical examination, sigmoidoscopy and biopsy, stool testing for microscopy, fecal calprotectin, and *Clostridiodes difficile*).

Apart from information about demographic and socioeconomic class, a detailed questionnaire was administered to the patients by an investigator well-versed in English and local languages (Hindi and Punjabi). We collected information about disease activity, duration, extent, currently prescribed medication, and socioeconomic status as per the Modified Kuppuswamy scale.

### Study questionnaire

The study questionnaire was prepared by the authors after an extensive review of previously published literature. After the initial drafting of the questionnaire, it was pre-tested in 2 male and female patients, each in Hindi as well as Punjabi languages. Therefore a total of 8 questionnaires were pretested, and a final questionnaire was prepared. The final questionnaire was administered by one investigator only (VSR), who was well-versed in Hindi and Punjabi. The questions were asked from the patients. The questions included an assessment of.

- Recent infections, including a preceding history suggestive of any upper respiratory infections, urinary tract infections, fever with skin rash.

- Recent use of any additional drugs like antibiotics, or pain medication (NSAIDs). This information was assessed after carefully reviewing the entire medical records of each individual patient.

- Lifestyle habits, including recent traveling, eating out, and any newly diagnosed comorbidities and changes in therapy.

- Stress, including if they felt stressed. This was based on a five-point Likert scale (1: no stress, 2: mild stress, 3: moderate stress, 4: much stress, 5: extreme stress). If they felt stressed (any score of > 1), an open-ended question about why they were stressed was asked. In case they were unable to provide an answer, possible reasons were enumerated and queried by the investigator.

- Non-adherence to prescribed therapies was also inquired, and they were further queried about the reasons for lack of adherence. This was again asked as an open-ended question followed by enumerating possible reasons if they could not provide an answer. Non-adherence was defined as stopping the drug altogether or missing any dose, irrespective of the duration. It was graded as missing the drug for either less than three days, 3–7 days, or more than 7 days.

- The use of alternative and complementary medication, including ayurvedic preparations was also enquired into.

- Any addiction and recent changes in the use of alcohol or smoking were enquired.

- The intake of milk, as well as consumption of milk products in the diet, was also ascertained.

- The final question was an open-ended query to the patients with flares on what they thought was a reason for flare.

### Statistical analysis

The statistical analysis was done using the SPSS version 28. The categorical data were reported as numbers and percentages and compared using Chi-square test. The continuous variables were reported as Median and interquartile range and compared using Mann Whitney U test as the data were non-parametric as per the Kolmogorov-Smirnov test.

## Results

### Baseline comparison

A total of 84 patients (51 with flare and 33 in remission) were enrolled from January 2022 to November 2022. The median age of the study group was 38 years (IQR: 24-49.3 years) and was similar between cases and controls (38 yrs, IQR: 23–50 and 38 yrs, IQR: 25.5–48.5; p- value-0.836). There were 47 (55.9%) males, which were similar between cases and controls (52.9% vs. 60.6%, p-value- 0.489). The disease extent between both the groups (41.2% with E3 and 15.7% with E2 in cases as compared to 21.2% with E3 and 21.2% with E2 in controls) was statistically similar.

The presence of underlying extraintestinal manifestations (21.6% vs. 12.1%, p-value- 0.269), comorbidities (31.4% vs. 27.3%, p-value- 0.688), smoking (11.8% vs. 3%%, p-value- 0.157) and alcohol use (15.7% vs. 15.2%, p-value- 0.947) were similar between both the groups. The use of 5-Aminosalicylates (76.5% vs. 90.9%, p-value 0.091) and advanced therapies, including biologicals and small molecules (1.9% vs. 3%, p-value- 0.754), was also similar between both the groups. However, the use of thiopurines was significantly higher in patients who were in remission as compared to those with flare (36.4% vs. 15.7%, p-value- 0.029). A total of 17 people reported taking antibiotics. Three of them did not know the name of the antibiotics they had taken. Amongst the remaining, Ofloxacin was the most common antibiotic used by six patients, followed by ciprofloxacin (2 patients), followed by ornidazole (5 patients, all in combination with ofloxacin), Cefixime (4) and Azithromycin (2). Table 1 shows the comparison of baseline parameters between cases and controls.

**Table 1:** Comparison of baseline characteristics between the cases (flare of ulcerative colitis) and controls (remission)

	Cases (n = 51) N(%)	Controls (n = 33)	p value
Age (years)	38 (24–49.3)	38 (25.5–48.53)	
Male gender	27 (52.9%)	20 (60.6%)	0.509
Marital status (Married)	35 (68.6%)	24 (72.7%)	0.377
Extent	E1- 1 (1.9%) E2- 8 (15.7%) E3- 21 (41.1%) UK- 21 (41.1%)	E1- 3 (9.1%) E2- 7 (21.2%) E3- 7 (21.2%) UK-16 (48.5%)	0.163
Extraintestinal manifestations	Yes- 11 (21.6%)	Yes − 4 (7.8%)	0.381
Comorbidity	Yes − 16 (31.4%)	Yes-9 (27.3%)	0.808
Smokers	6 (11.8%)	1 (3%)	0.237
Alcohol use	8 (15.7%)	5 (15.2)	1.00
5 Aminosalicylates use	39 (76.5%)	30 (90.9%)	0.144
**Thiopurine use**	**8 (15.7%)**	**12 (36.36%)**	**0.038**
Biologics/small molecule use	1 (1.9%)	1 (3%)	1.0
Kuppuswamy scale (median)	10	8	0.25

### Predictors of Flare

A total of 20 patients (39.2%) of patients with flare, as compared to 10 patients (30.3%) of those in remission, had a history of recent infection in the previous three months, and these were statistically similar (p-value 0.405). The history of recent travel was similar between both the groups (31.4% vs. 27.3%, p-value- 0.688). The history of eating out was similar between the groups (47.1% vs. 39.4%, p-value- 0.489). While 88.2% of those with flare consumed milk products, 75.8% of those in remission reported consuming milk products (p = 0.134). Recent use of pain medication was reported by 43.1% of those with a flare compared to 33.3% amongst those in remission (p = 0.369). Most patients in both groups reported a history of recent stress (62.7% in cases and 60.6% in controls, p-value- 0.844).

The groups had differences in the recent intake of drugs with a higher intake of antibiotics (29.4% in cases vs. 6.1% in controls, p-value − 0.011). Lack of adherence to therapy, either by stopping the drug or missing drug doses, was higher amongst those who had a flare (50.9% vs. 15.2%, p-value < 0.001). The intake of complementary and alternative medication was also higher in those having a disease flare (70.6% vs. 33.3%, p-value < 0.001) (Table 2). Forty-seven patients took alternative treatment in total. Out of these, 23 took it after discontinuing IBD therapy, while 24 took it as an adjunct.

**Table 2:** Comparison of possible predictors of flares characteristics between the cases (flare of ulcerative colitis) and controls (remission)

Predictors of flare	Cases (n = 51)	Controls (n = 33)	p value
Infection	Yes − 20 (39.2%)	Yes-10 (30.3)	0.405
Recent travel	Yes − 16 (31.4%)	Yes − 9 (27.3%)	0.688
Eating outside food	Yes − 24 (47.1%)	Yes − 13 (39.4%)	0.489
Milk products	Yes- 45 (88.2%)	Yes − 25 (75.8%)	0.134
Pain killers	Yes-22 (43.1%)	Yes-11 (33.3%)	0.369
Recent stress	Yes − 32 (62.7%)	Yes − 20 (60.6%)	0.844
**Lack of adherence**	**Yes-26 (50.9%)**	**Yes − 5 (15.2%)**	**< 0.001**
**CAM use**	**Yes-36 (70.6%)**	**Yes-11 (33.3%)**	**< 0.001**
**Antibiotic use**	**Yes-15 (29.4%)**	**Yes- 2 (6.1%)**	**0.011**

### Perceptions in patients with flares

Some of the questions were exploratory in an attempt to understand the reasons behind the flares. Regarding pain medication use, 12 out of 23 patients (52.2%) took nonsteroidal anti-inflammatory drugs (NSAIDs), 1 (4.3%) took non-NSAIDs (Acetaminophen as well as narcotic drugs like Opioids) and the rest did not remember which drug was used. The reasons for intake of pain medication were generalized body aches (17.6%), pain abdomen (13.7%) and less frequently, fever (3.9%), headache (3.9%), symptomatic gallstone disease (1.9%), knee pain (1.9%) and leg pain (1.9%).

Thirty-two (62.7%) patients with disease flare reported a history of recent stress. Disease-related symptoms (35.3%) were the major cause of stress amongst patients with flare. Job-related stress (11.7%), separation from family (3.9%), and recent bereavement in the family (3.9%) were the other main reasons for stress. A small proportion of patients also reported financial reasons (5.8%), drug-related alopecia (1.9%), children’s marriage (3.9%), and busy academic schedule (3.9%) as the reason for their recent stress before having a flare. One MBBS student studying in Ukraine cited the Ukraine- Russia conflict resulting in the disruption of his medical education as the reason for his stress.

Twenty-six (50.9%) patients amongst those with flare reported a lack of adherence to therapy before the onset of flare. Fifteen (57.7%) patients stopped their drugs for more than ten weeks while 11 (42.3%) patients did so for less than ten weeks before the onset of flare. The cost of the drugs (38.5%) was the major factor that led to the stoppage of drugs by the patients themselves. Presumed cure of disease (26.9%) due to lack of symptoms and medication-related adverse effects (26.9%) were the other main reasons for stopping drugs. Most patients with flare had a history of complementary and alternative medicine intake (36 patients, 70.6%). Sixteen (44.4%) patients took CAM for 1–4 weeks, 9 (25%) patients took for 5–16 weeks, while 8 (22.2%) patients took CAM for > 16 weeks. The main reasons for starting CAM among patients with disease flare were a suboptimal response to the current treatment (21.5%) and promised ‘permanent cure’ (13.7%). Other reasons were additional benefits (5.9%), and other diseases (7.8%).

Forty-five (88.2%) patients had certain perceptions regarding the cause of their flare. Most of these patients (42.2%) believed that faulty eating habits, including eating outside food, was the reason for their relapse. Noncompliance to drugs (22.2%), recent stress (11.1%), CAM intake (2.2%) were other perceived reasons for the cause of their relapse. Interestingly, a few patients attributed their disease flare to “Karma”, stress from a Google search which showed including cancer as a possible complication of the disease, COVID vaccination, and COVID lockdown-related lack of medication access.

The socio economic status of all patients was assessed using the modified Kuppuswamy scale. The median modified Kuppuswamy scale was 10 amongst controls as compared to 8 amongst cases. The correlation between Kuppuswamy scale between cases and controls was not statistically significant (p value 0.25).

## Discussion

The present study at a single center in North India provides novel and actionable insights into the reasons for flare in patients with ulcerative colitis. The case-control design helped identify factors like lack of adherence to therapies and use of complementary and alternative therapies as possible reasons for flare. Understandably, thiopurine use was higher in patients with remission. There were unaddressed symptoms like pain which necessitated pain medication intake in a significant number in the two groups, and patient-perceived stress was equally frequent in the two groups. These results provide grounds for future work on understanding the psychological and economic aspects of chronic diseases like UC, which may result in a lack of adherence and thereby result in flares.

Chronic diseases are recognized to be associated with non-adherence to therapies. The reasons are manifold and include costs of ongoing therapy, lack of response, complete response (which is thought to be a cure), and concerns about adverse effects [18]. Medical non-adherence is also well-recognized in inflammatory bowel disease [19]. Studies have reported varying factors for the prediction of non-adherence, including female gender, educational level, type of IBD, distal disease, disability, smoking, and age [19–21]. In India, the easy availability of unregulated complementary and alternative medication proclaiming ‘cure’ and ‘magical outcomes’ adds another dimension to the non-adherence [22]. Even in the West, many IBD patients report using CAM at some point [23, 24]. Some studies suggest that the lack of benefits with modern medicine may also be an important factor in patients using CAM. Educational interventions which inform patients about the response rates of IBD therapies, adverse effects and safety, and expected improvement (or lack of improvement) may help reduce non-adherence rates [25]. The costs of IBD therapies remain a significant barrier, and the cost of 5-aminosalicylates in India is extremely high as compared to thiopurines [1]. A large study done in Sweden recently linked antibiotic use to the onset of flare in patients with ulcerative colitis [26]. Their indiscriminate use in managing acute diarrhea and over-the-counter availability in India is an important factor leading to flares [27].

Recent literature suggests additional insights into the cause of flares in IBD. Proposed factors include visceral adiposity, the use of conventional or no therapy, polypharmacy [27–30]. On the other hand use of COVID vaccination is not associated with IBD flares [31]. Only very few studies report about the risk factors of flare from India. In a study by Dhingra et al., intake of vitamin A and recent NSAID use predicted flares. This study reported on psychological factors and dietary influences in causation of flare but nonadherence was not reported as an important factor. The possible reason could be the inclusion of patients who were following up regularly with the clinic and reported for visits every three months [31].

Our study has some limitations- the results are based on a patient interview which may have a recall bias, and the study has been conducted at a single center. Since we only included patients with ulcerative colitis, the results may not be extrapolated to all patients with IBD. Further, the estimation of stress was based on patient perception, which may be fallacious. In addition, since the study was conducted during the COVID pandemic with disruptions in OPD, we did not formally calculate sample size a priori. The limited number of patients with remission is another limitation. Also, our study may not reflect the causes of flares in the Western world as the use of biologics and small molecules is limited in our population. We did not specifically look for illegal drug use like marijuana. Further, factors such as antibiotic use, nonadherence to medications, and CAM intake could be associated with the disease process as such and these may not be true precipitates in actual sense. In view of the cross-sectional nature of this study, it was not possible to evaluate the disease state prior to exposure to these factors.

However, there are certain strengths of the study including the quantitative evaluation of patient perceptions which brought out novel and interesting issues. Further the case-control design helped discriminate certain factors like stress which although was common in those with flares but also high in those with remission. We believe that the results of this study provide ground for future work on education interventions regarding the IBD therapies to improve the adherence rates for IBD therapies.

To conclude, the present study identified lack of adherence to IBD therapies and use of antibiotics and CAM as possible precipitants of flare in ulcerative colitis. Future studies should attempt to address these issues using educational intervention to avoid flares.

### Electronic Supplementary Material

Below is the link to the electronic supplementary material

Supplementary Material 1

## Data Availability

The data can be provided by the corresponding author on reasonable request.
