# Supplementary material for: Factors contributing to flares of ulcerative colitis in North India- a case-control study
Source: BMC Gastroenterol. 2023 Sep 28;23:336. doi: 10.1186/s12876-023-02978-y (PMC10540407; doi:10.1186/s12876-023-02978-y)
Supplement: Supplementary file 1 — Supplementary Material 1 [file 12876_2023_2978_MOESM1_ESM.pdf]

SCREENING PROFORMA

NAME Age: Gender:

CR Number Date of Presentation:

Phone.no: Mobile No:

REASON FOR ADMISSION

1]FLARE- NON SEVERE

2] FLARE- ACUTESEVEREULCERATIVECOLITIS

3] EXTRAINTESTINALMANIFESTATION

4]] OTHERS

SCCAI

Bowel frequency during day	1–3	4–6	7–9		
Bowel frequency during night	0-01–3 1				
Urgency of defecation	Hurry	Immediately	Incontinence		
**Blood in stool**	Trace	Usually frank	Occasionally frank		
**General wellbeing**	Very well	Slightly below par	Poor	Very poor	Terrible

Extracolonic features

1 per manifestation

Total score-

Fecal calprotectin-

Eligible for inclusion as CASE/ Control/ Not ELIGIBLE

CASE RECORD FORM

NAME CR Number

Date of Presentation: Place:

Phone.no: Mobile No:

Age: Gender:

Residential address :

State District Village Category

FLARE/REMISSION: Date of diagnosis:

Married/ Single :

Joint/Nuclear family/Alone:

SES Status: Modified Kuppuwamy scale

Extent: E1/E2/E3 /uncharacterized

Extraintestinal manifestations: Yes……./No………

Musculoskeletal…………………../ Yes……./No………Ocular…………./Yes……./No………

Dermatological…………………./Yes……./No………

Hepatobiliary/pancreatic………………../Yes……./No………

Bronchopulmonary ………………Yes……./No………

Hematologic …………………./Yes……./No………

Endocrine…………………../Yes……./No………

Reason for admission

A] Flare (nonsevere)

B] Flare (acute severe UC)

C] Extraintestinal Manifestation D] Other

Duration of hospital stay

List of Prescribed drugs-

List of drugs currently taking

Questionnaire (This is an English translation of the questionnaire from Hindi/Punjabi. The questionnaire used was in the vernacular languages)

1. Did you had any kind of infection or infection in the last 3 months? (check any medical records)

2. Did you have any of the following symptoms in the last 3 months

a. Cough, sputum

b. Burning in urine

c. Any mark or spots on the skin

d. Diarrhea

3. If you had an infection, when did it happen?

4. Have you used any antibiotic in the last 3 months?

5. If yes, which antibiotic (Check records)

6. Have you traveled outside your village or city in the last one month?

7. If yes, where _____________

8. Have you eaten outside in the last 1 month

9. If yes then from where______________

10. Have you consumed any milk products in the last 2 weeks

11. If yes, which milk products

12. Have you used any pain-relieving medication in the last 3 months

13. If yes then which medicine (Check records)

14. If you used pain medication, why was it needed?

15. Do you have any other diseases besides IBD?

16. Are you taking any medicine (Check list)

17. Has there been any change in any of your medicines in the last 3 months

18. Have you been under any stress in the last 3 months?

No Stress - 1

Mild Stress - 2

Moderate Stress - 3

Much Stress - 4

Extreme Stress – 5

19. If you were under stress, what was the reason?

a. Divorce or Separation

b. Due to illness

c. Due to a death in the family

d Due to change in job or loss of job

e. Due to the birth of a child

f. Stress in your work

g. Any other reason (please specify)

20. Have you taken any Ayurvedic, Homeopathic or Herbal medicine in the last 3 months?

21. If you have used such medicine, please tell why you used the medicine and for how long

22. Have you stopped your ulcerative colitis medicine?

23. If yes, for how long

24. What is the reason behind stopping the medicine

a. Medicines being expensive

b. Due to any harm or side effects from the medicine

c. Due to fear of harm from medicine

d. Due to no effect from the medicine

e. Any other reason

25. Have you missed a dose of medicine?

26. If yes, for what period

a. Less than 3 days in a month

b. Between 3 to 7 days

c. More than 1 week in a month

27. Do you consume alcohol?

a. If yes, for how long

b. How much do you consume per day

c. When was the last time you consumed alcohol?

28. Do you smoke?

a. If yes, for how long

b. How much do you consume per day

c. When was the last time you smoked

29. Do you do any other kind of intoxicant? if so whose

30. In your opinion, what is the reason for your aggravation at this time?

a. Stopping the medicine

b By taking indigenous or Ayurvedic medicines

c. Due to improper eating

d. Other reasons (please specify)

ਪ੍ਰਸ਼ਨਾਵਲੀ

1. ਕੀ ਤੁਹਾਨੂੰ ਪਿਛਲੇ 3 ਮਹੀਨਿਆਂ ਵਿੱਚ ਕਿਸੇ ਕਿਸਮ ਦੀ ਲਾਗ ਜਾਂ ਸੰਕਰਮਣ ਹੋਇਆ ਹੈ? (ਕਿਸੇ ਵੀ ਮੈਡੀਕਲ ਰਿਕਾਰਡ ਦੀ ਜਾਂਚ ਕਰੋ)
2. ਤੁਹਾਨੂੰ ਪਿਛਲੇ 3 ਮਹੀਨਿਆਂ ਵਿੱਚ ਹੇਠ ਲਿਖੇ ਲੱਛਣਾਂ ਵਿੱਚੋਂ ਕੋਈ ਵੀ ਮਿਲਿਆ ਹੈ

ਖੰਘ, ਬਲਗਮ

ਡਾਇਸੂਰੀਆ

ਚਮੜੀ ਧੱਫੜ

ਦਸਤ

3. ਜੇਕਰ ਤੁਹਾਨੂੰ ਕੋਈ ਲਾਗ ਸੀ, ਤਾਂ ਇਹ ਕਦੋਂ ਹੋਇਆ?
4. ਕੀ ਤੁਸੀਂ ਪਿਛਲੇ 3 ਮਹੀਨਿਆਂ ਵਿੱਚ ਕੋਈ ਐਂਟੀਬਾਇਓਟਿਕਸ ਲਏ ਹਨ?
5. ਜੇਕਰ ਹਾਂ, ਤਾਂ ਕਿਹੜੀ ਐਂਟੀਬਾਇਓਟਿਕ (ਰਿਕਾਰਡ ਦੀ ਜਾਂਚ ਕਰੋ)
6. ਕੀ ਤੁਸੀਂ ਪਿਛਲੇ ਇੱਕ ਮਹੀਨੇ ਵਿੱਚ ਆਪਣੇ ਪਿੰਡ ਜਾਂ ਸ਼ਹਿਰ ਤੋਂ ਬਾਹਰ ਯਾਤਰਾ ਕੀਤੀ ਹੈ?
7. ਜੇਕਰ ਹਾਂ ਤਾਂ ਕਿੱਥੇ ______________
8. ਕੀ ਤੁਸੀਂ ਪਿਛਲੇ 1 ਮਹੀਨੇ ਵਿੱਚ ਬਾਹਰ ਖਾਧਾ ਹੈ?
9. ਜੇ ਹਾਂ, ਤਾਂ ਕਿੱਥੋਂ ________________
10. ਕੀ ਤੁਸੀਂ ਪਿਛਲੇ 2 ਹਫ਼ਤਿਆਂ ਵਿੱਚ ਕੋਈ ਵੀ ਡੇਅਰੀ ਉਤਪਾਦ ਖਾਧਾ ਹੈ?
11. ਜੇਕਰ ਅਜਿਹਾ ਹੈ ਤਾਂ ਕਿਹੜੀ ਸਮੱਗਰੀ
12. ਕੀ ਤੁਸੀਂ ਪਿਛਲੇ 3 ਮਹੀਨਿਆਂ ਵਿੱਚ ਕੋਈ ਦਰਦ ਨਿਵਾਰਕ ਦਵਾਈਆਂ ਦੀ ਵਰਤੋਂ ਕੀਤੀ ਹੈ?
13. ਜੇਕਰ ਹਾਂ ਤਾਂ ਕਿਹੜੀ ਦਵਾਈ (ਰਿਕਾਰਡ ਚੈੱਕ ਕਰੋ)
14. ਜੇ ਤੁਸੀਂ ਦਰਦ ਨਿਵਾਰਕ ਦਵਾਈਆਂ ਦੀ ਵਰਤੋਂ ਕਰਦੇ ਹੋ, ਤਾਂ ਇਸਦੀ ਲੋੜ ਕਿਉਂ ਸੀ?
15. ਕੀ ਤੁਹਾਨੂੰ IBD ਤੋਂ ਇਲਾਵਾ ਹੋਰ ਬਿਮਾਰੀਆਂ ਹਨ?
16. ਕੀ ਤੁਸੀਂ ਕੋਈ ਦਵਾਈ ਲੈ ਰਹੇ ਹੋ (ਸੂਚੀ ਦੀ ਜਾਂਚ ਕਰੋ)
17. ਕੀ ਪਿਛਲੇ 3 ਮਹੀਨਿਆਂ ਵਿੱਚ ਤੁਹਾਡੀ ਕਿਸੇ ਦਵਾਈ ਵਿੱਚ ਕੋਈ ਬਦਲਾਅ ਹੋਇਆ ਹੈ?
18. ਕੀ ਤੁਸੀਂ ਪਿਛਲੇ 3 ਮਹੀਨਿਆਂ ਵਿੱਚ ਕਿਸੇ ਤਣਾਅ ਵਿੱਚ ਰਹੇ ਹੋ?

ਬਿਲਕੁਲ ਨਹੀਂ ਬੀ. ਥੋੜਾ ਬਹੁਤ ਜ਼ਿਆਦਾ ਤਣਾਅ c. ਤਣਾਅ ਹੈ ਡੀ. ਬਹੁਤ ਜ਼ਿਆਦਾ ਤਣਾਅ ਈ. ਬਹੁਤ ਜ਼ਿਆਦਾ ਤਣਾਅ

19. ਜੇਕਰ ਤੁਸੀਂ ਤਣਾਅ ਵਿੱਚ ਸੀ, ਤਾਂ ਇਸਦਾ ਕਾਰਨ ਕੀ ਸੀ

ਤਲਾਕ ਜਾਂ ਵੱਖ ਹੋਣਾ

ਬਿਮਾਰੀ ਦੇ ਕਾਰਨ

ਪਰਿਵਾਰ ਵਿੱਚ ਇੱਕ ਮੌਤ ਦੇ ਕਾਰਨ

ਨੌਕਰੀ ਬਦਲਣ ਜਾਂ ਨੌਕਰੀ ਦੇ ਨੁਕਸਾਨ ਕਾਰਨ

ਬੱਚੇ ਦੇ ਜਨਮ ਦੇ ਕਾਰਨ

ਕੰਮ 'ਤੇ ਤਣਾਅ

ਕੋਈ ਹੋਰ ਕਾਰਨ (ਕਿਰਪਾ ਕਰਕੇ ਦੱਸੋ)

20. ਕੀ ਤੁਸੀਂ ਪਿਛਲੇ 3 ਮਹੀਨਿਆਂ ਵਿੱਚ ਕੋਈ ਆਯੁਰਵੈਦਿਕ, ਹੋਮਿਓਪੈਥਿਕ ਜਾਂ ਹਰਬਲ ਦਵਾਈ ਲਈ ਹੈ?
21. ਜੇਕਰ ਤੁਸੀਂ ਅਜਿਹੀ ਦਵਾਈ ਦੀ ਵਰਤੋਂ ਕੀਤੀ ਹੈ, ਤਾਂ ਕਿਰਪਾ ਕਰਕੇ ਦੱਸੋ ਕਿ ਦਵਾਈ ਕਿਸ ਲਈ ਅਤੇ ਕਿੰਨੇ ਸਮੇਂ ਲਈ ਵਰਤੀ ਗਈ ਸੀ
22. ਕੀ ਤੁਸੀਂ ਆਪਣੀ ਅਲਸਰੇਟਿਵ ਕੋਲਾਈਟਿਸ ਦੀ ਦਵਾਈ ਬੰਦ ਕਰ ਦਿੱਤੀ ਹੈ?
23. ਜੇਕਰ ਅਜਿਹਾ ਹੈ ਤਾਂ ਕਿੰਨੇ ਸਮੇਂ ਲਈ
24. ਦਵਾਈ ਬੰਦ ਕਰਨ ਦਾ ਕੀ ਕਾਰਨ ਹੈ

ਦਵਾਈ ਦੀ ਲਾਗਤ

ਦਵਾਈ ਦੇ ਕਿਸੇ ਵੀ ਨੁਕਸਾਨ ਜਾਂ ਮਾੜੇ ਪ੍ਰਭਾਵਾਂ ਦੇ ਕਾਰਨ

ਦਵਾਈ ਤੋਂ ਨੁਕਸਾਨ ਦੇ ਡਰ ਕਾਰਨ

ਦਵਾਈ ਦਾ ਕੋਈ ਜਵਾਬ ਨਾ ਮਿਲਣ ਕਾਰਨ

ਕੋਈ ਹੋਰ ਕਾਰਨ

25. ਕੀ ਤੁਸੀਂ ਦਵਾਈ ਦੀ ਇੱਕ ਖੁਰਾਕ ਛੱਡੀ ਦਿੱਤੀ ਹੈ
26. ਜੇਕਰ ਹਾਂ, ਤਾਂ ਕਿਸ ਮਿਆਦ ਲਈ

ਇੱਕ ਮਹੀਨੇ ਵਿੱਚ 3 ਦਿਨਾਂ ਤੋਂ ਘੱਟ

3 ਤੋਂ 7 ਦਿਨਾਂ ਦੇ ਵਿਚਕਾਰ

ਇੱਕ ਮਹੀਨੇ ਵਿੱਚ 1 ਹਫ਼ਤੇ ਤੋਂ ਵੱਧ

27. ਕੀ ਤੁਸੀਂ ਸ਼ਰਾਬ ਪੀਂਦੇ ਹੋ?

ਜੇਕਰ ਅਜਿਹਾ ਹੈ ਤਾਂ ਕਿੰਨੇ ਸਮੇਂ ਲਈ

ਤੁਸੀਂ ਪ੍ਰਤੀ ਦਿਨ ਕਿੰਨਾ ਪੀਂਦੇ ਹੋ

ਤੁਸੀਂ ਆਖਰੀ ਵਾਰ ਕਦੋਂ ਸ਼ਰਾਬ ਪੀਤੀ ਸੀ

28. ਕੀ ਤੁਸੀਂ ਧੂਮਰਪਾਨ ਕਰਦੇ ਹੋ

ਜੇਕਰ ਅਜਿਹਾ ਹੈ ਤਾਂ ਕਿੰਨੇ ਸਮੇਂ ਲਈ

ਤੁਸੀਂ ਪ੍ਰਤੀ ਦਿਨ ਕਿੰਨਾ ਪੀਂਦੇ ਹੋ

ਤੁਸੀਂ ਆਖਰੀ ਵਾਰ ਕਦੋਂ ਸਿਗਰਟ ਪੀਤੀ ਸੀ

29. ਕੀ ਤੁਸੀਂ ਕਿਸੇ ਹੋਰ ਕਿਸਮ ਦੇ ਨਸ਼ੇ ਕਰਦੇ ਹੋ? ਜੇਕਰ ਅਜਿਹਾ ਹੈ ਤਾਂ ਕਿਸਦਾ
30. ਤੁਸੀਂ ਕੀ ਸੋਚਦੇ ਹੋ ਕਿ ਇਸ ਸਮੇਂ ਤੁਹਾਡੀ ਬਿਮਾਰੀ ਵਧ ਰਹੀ ਹੈ?

ਡਰੱਗ ਕਢਵਾਉਣਾ

ਦੇਸੀ ਜਾਂ ਆਯੁਰਵੈਦਿਕ ਦਵਾਈਆਂ ਲੈ ਕੇ

ਗਲਤ ਖਾਣ ਦੇ ਕਾਰਨ

ਹੋਰ ਕਾਰਨ (ਕਿਰਪਾ ਕਰਕੇ ਦੱਸੋ)

1. क्या आपको पिछले 3 महीने में किसी प्रकार का संक्रमण अथवा इंफेक्शन हुआ था ? (check any medical records)
2. पिछले 3 महीने में आपको निम्नलिखित में से कोई भी लक्षण हुआ था
    1. खांसी, बलगम
    2. पेशाब में जलन
    3. चमड़ी पर किसी किस्म का निशान या धब्बे
    4. दस्त लगना
3. यदि आपको कोई इंफेक्शन हुआ था तो यह कब हुआ
4. क्या आपने पिछले 3 महीने में किसी एंटीबायोटिक का सेवन किया है
5. यदि हां, तो किस एंटीबायोटिक का (Check records)
6. क्या आप पिछले एक महीने में अपने गांव या शहर के बाहर कहीं गए थे
7. अगर हां तो कहां _____________
8. क्या आपने पिछले 1 महीने में घर के बाहर भोजन किया है
9. यदि हां तो कहां से______________
10. क्या आपने पिछले 2 हफ्तों में किसी भी दूध से बने पदार्थ का सेवन किया है
11. यदि हां तो किस पदार्थ का
12. क्या आपने पिछले 3 महीने में किसी भी दर्द निवारक दवाई का इस्तेमाल किया है
13. यदि हां तो किस दवाई का (Check records)
14. यदि आपने दर्द निवारक दवाई का इस्तेमाल किया है तो इसकी आवश्यकता क्यों पड़ी
15. क्या IBD के अलावा आपको और भी कोई अन्य बीमारी है
16. क्या आप कोई भी दवाई का सेवन कर रहे हैं (Check list)
17. क्या पिछले 3 महीने में आपकी किसी भी दवाई में कोई परिवर्तन हुआ है
18. क्या आप पिछले 3 महीने में किसी भी अथवा तनाव में रहे हैं
    1. बिल्कुल नहीं b. थोड़ा बहुत तनाव c. तनाव हुआ है d. काफी तनाव e. अत्यधिक तनाव
19. यदि आप तनाव में थे, तो उसका क्या कारण था
    1. तलाक अथवा अलगाव
    2. बीमारी के कारण
    3. परिवार में किसी मृत्यु के कारण
    4. नौकरी में बदलाव या नौकरी छूटने के कारण
    5. बच्चे के जन्म के कारण
    6. अपने कार्य में तनाव
    7. कोई अन्य कारण (कृपया बताएं)
20. क्या आपने पिछले 3 महीने में किसी भी आयुर्वेदिक, होम्योपैथिक अथवा हर्बल दवाई का सेवन किया है

21. यदि आपने ऐसी दवाई का इस्तेमाल किया है तो कृपया बताएं की दवाई का सेवन किस लिए किया और कितनी अवधि के किया
22. क्या आपने अपनी अल्सरेटिव कोलाइटिस की दवाई बंद की
23. यदि हां तो कितने समय से
24. दवाई बंद करने के पीछे क्या कारण है
    1. दवाई महंगी होना
    2. दवाई से किसी हानि अथवा खराब असर के कारण
    3. दवाई से नुकसान के डर के कारण
    4. दवाई से फर्क नहीं पड़ने के कारण
    5. कोई अन्य कारण
25. क्या आपने दवाई कि कुछ खुराक छोड़ी है
26. यदि हां तो कितनी अवधि के लिए
    1. महीने में 3दिन से कम
    2. 3 से 7 दिन के बीच में
    3. महीने में 1 हफ्ते से ज्यादा
27. क्या आप शराब का सेवन करते हैं
    1. यदि हां तो कितने समय से
    2. प्रतिदिन आप कितना सेवन करते हैं
    3. अपने आखिरी बार शराब का सेवन कब किया था
28. क्या आप धूम्रपान करते हैं
    1. यदि हां तो कितने समय से
    2. प्रतिदिन आप कितना सेवन करते हैं
    3. अपने आखिरी बार धूम्रपान का सेवन कब किया था
29. क्या आप किसी अन्य किस्म का नशा करते हैं? यदि हां तो किसका
30. आपके अनुमान से, इस समय आप की बीमारी बढ़ने का क्या कारण है
    1. दवाई बंद करना
    2. देसी अथवा आयुर्वेदिक दवाइयां खाने से
    3. अनुचित खाने के कारण
    4. अन्य कारण (कृपया बताएं)
